# Balsa Wood-Loaded Polyvinyl Alcohol/Chitosan/Zinc Gluconate Hydrogel Applied as Wound Dressing

**DOI:** 10.3390/polym18131677

**Published:** 2026-07-07

**Authors:** HanJiong Ji, Shengqiang Liao, Shibo Wu, Sijia Chen, Xue Guan, Chenlong Li, Dawei Zhang

**Affiliations:** 1 Aulin College, Northeast Forestry University, Harbin 150040, China; jihanjiong116@163.com; 2 State Key Laboratory of Woody Oil Resources Utilization, Northeast Forestry University, Harbin 150040, China; 2021210384@nefu.edu.cn (S.L.); s548_4@163.com (S.W.); ciga0623@163.com (S.C.); 3 Animal Laboratory Center, The Second Affiliated Hospital of Harbin Medical University, Harbin 150081, China; guanxue2019@163.com; 4 Department of Neurosurgery, Harbin Medical University Cancer Hospital, Nangang District, Harbin 150086, China

**Keywords:** balsa wood, polyvinyl alcohol, chitosan, zinc gluconate, antibacterial properties, wound dressing

## Abstract

The skin is the largest organ of the human body and, due to its direct contact with the external environment, is one of the most vulnerable tissues. Traditional medical bandages and gauze exhibit limited efficacy in wound management, often neglecting the control of wound inflammation and the promotion of skin regeneration. Hydrogels, as an emerging material, possess appropriate swelling capacity, oxygen permeability, and the ability to absorb wound exudates, thereby facilitating wound healing, making them an ideal choice for functional applications in skin tissue engineering. In this study, dual-treated balsa wood (BWSM) was used as the hydrogel substrate, with polyvinyl alcohol (PVA), chitosan (CS), and zinc gluconate (ZnG) used as the primary raw materials. The BWSM/PVA/CS/ZnG hydrogel was prepared via gamma-ray irradiation. Balsa wood treated with alkaline solutions, hydrogen peroxide solutions, and microwave treatment processing exhibited enhanced transparency, increased porosity, improved thermal stability and swelling rates, while retaining adequate mechanical strength. Gamma-ray irradiation of the BWSM/PVA/CS/ZnG hydrogel wound dressing demonstrated sustained drug release and antibacterial efficacy through release and antimicrobial tests. Animal experiments showed that the BWSM/PVA/CS/ZnG composite hydrogel promoted wound healing in mice and effectively prevented scar formation. The aforementioned results demonstrate that the PVA/CS/ZnG composite hydrogel loaded with balsa wood exhibits durable antibacterial properties and high mechanical strength and promotes wound healing, making it suitable for applications in biomedical materials such as wound dressings.

## 1. Introduction

The skin is the largest organ of the human body, primarily composed of the epidermis—a thin layer responsible for resisting mechanical, chemical, or biological stimuli—and the dermis, which is typically thicker than the epidermis and contains fibroblasts and an extracellular matrix. Due to its direct contact with the external environment, the skin is also one of the most vulnerable tissues. Wounds have historically posed significant challenges and remain a substantial burden on healthcare systems. In 2014, wounds affected over 8 million individuals in the United States, with estimated economic losses reaching $30 billion. With the aging population and rising obesity rates, high-risk comorbidities have correspondingly increased; the market size for wound closure products reached $21.4 billion in 2022, with a compound annual growth rate of 4.15% from 2023 to 2030 [1,2]. Normally, healthy skin initiates a series of synchronized healing processes following wound formation, including hemostasis, inflammation, re-epithelialization, and tissue remodeling, during which coagulation factors, inflammatory cells and cytokines, growth factors, and other elements collectively contribute to wound closure. Consequently, wound healing may be prone to obstruction due to the complexity of the process and the presence of underlying comorbidities such as infections, diabetes, ischemia, and malnutrition. However, traditional medical bandages and gauze demonstrate limited efficacy in wound management. While hemostasis and infection prevention are achieved through bandages and gauze, these approaches overlook the management of wound inflammation and the promotion of skin regeneration, making them increasingly inadequate for clinical applications [3,4].

Hydrogels, a type of three-dimensional networked polymer material, contain numerous hydrophilic groups and pores, enabling them to retain substantial amounts of water and fluid substances through swelling [5]. Hydrogels offer multiple advantages: appropriate swelling capacity, oxygen permeability, and the ability to absorb wound exudates, thereby promoting wound healing [6,7]. Due to their edematous and porous structural matrix, hydrogels provide a moist environment for the wound bed, facilitating cell proliferation and the absorption of wound exudates. Additionally, hydrogels possess considerable mechanical strength, resembling human skin, effectively protecting the wound site from pathogenic microorganisms and harsh external conditions. The self-healing properties of hydrogels also ensure their integrity and fatigue resistance, even when applied to joint regions. Beyond these inherent benefits, hydrogels can be further incorporated or modified with therapeutic agents such as inorganic materials, metal–organic frameworks, natural products, clinical drugs, growth factors, extracellular vesicles, and nanomaterials for targeted wound treatment. Once integrated, the hydrogel matrix exhibits diverse therapeutic effects, including antioxidant, antibacterial, anti-inflammatory, reactive oxygen species scavenging, immunomodulatory, glucose-regulating, and oxygen-supplementing properties, making it an excellent candidate for wound therapy. Consequently, hydrogels are regarded as an ideal choice for functional applications in skin tissue engineering [8,9,10,11].

In recent years, hydrogel systems fabricated through gamma-ray irradiation have garnered significant attention. When aqueous polymer solutions are irradiated, free radicals form along the polymer chains, while water molecules undergo radioactive decomposition to generate hydroxyl radicals. These hydroxyl radicals subsequently interact with the polymer chains to form macroradicals. These macroradicals then reorganize across different chains, establishing covalent bonds and ultimately forming a cross-linked structure. Hydrogels produced via this irradiation method require neither chemical initiators nor cross-linking agents; instead, gamma-ray irradiation enables active radical polymerization, offering multiple advantages for practical applications. Key benefits include easy controllability, environmental friendliness, the ability to synthesize and sterilize hydrogels in a single step while maintaining room temperature, and high permeability [12,13].

Balsa wood (BW) has a density of only 0.1 g/cm^3^, equivalent to one-tenth the weight of an equal volume of water. Despite its light weight, balsa wood possesses a tightly structured composition and excellent biocompatibility. After specific treatment, it exhibits high transparency and adequate mechanical strength. The lignin within its cell walls dissolves in strong alkaline solutions, where hydroxide ions react with phenolic hydroxyl groups in the lignin, neutralizing them and thereby opening the crystalline regions to create a porous structure. Subsequent microwave treatment further enhances the wood’s porosity, enabling its use as a substrate for hydrogel dressings [14,15,16,17].

Among various naturally occurring biopolymers, chitosan (CS) is the second most abundant, widely used, and naturally occurring biopolymer, synthesized through chitin deacetylation [18,19]. Chitosan exhibits excellent biocompatibility, regenerability, and biodegradability by human enzymes. It possesses the ability to activate anti-inflammatory cells (including polymorphonuclear leukocytes, lymphocytes, and macrophages) and promotes wound healing. Additionally, chitosan shares a structure and antibacterial activity similar to that of the extracellular matrix, enhancing its role in skin tissue engineering. Consequently, CS-based hydrogels hold significant application potential in various biomedical fields such as wound dressings, ulcer healing, and tissue scaffolds [20,21,22]. Polyvinyl alcohol (PVA) is a high-molecular-weight biopolymer composed of carbon chains and hydroxyl functional groups. Its notable characteristics include outstanding film-forming capability, structural flexibility, hydrophilicity, and biocompatibility. PVA forms a highly water-rich network through cross-linking, effectively mimicking the extracellular matrix environment [23,24]. Zinc gluconate (ZnG), as an organically derived zinc source with excellent biocompatibility, enables localized zinc supplementation at wound sites in zinc-containing medical dressings. These dressings effectively promote the healing of burns, trauma, surgical wounds, ulcers, and skin inflammation. The carboxyl and hydroxyl groups in their molecular structure form abundant hydrogen bonds and ion exchange cross-linking points with polymer chains (e.g., –OH in PVA or –NH_2_ in CS). This non-covalent interaction not only significantly improves the thermal decomposition temperature and micro-pore structure of the composite hydrogel, but also endows the material with sustained zinc ion (Zn^2+^) release capability, thereby exerting antibacterial effects while promoting cell proliferation [25,26].

Traditional hydrogel dressings, while providing a moist healing interface, are generally limited by mechanical weakness, rigid water management of exudate, lack of endogenous bioactivity, and the risk of damage associated with secondary fixation. Incorporating a PVA/CS active gel layer onto a balsa wood honeycomb cellulose scaffold leverages the wood’s natural high porosity and oriented microtubular channels to simultaneously achieve exudate buffering, dimensional stability, and tear-resistant support, elevating the dressing from a mere “interface material” to an integrated interface + load-bearing + storage/release platform [27,28]. In this architecture, zinc gluconate is selected as the zinc source because it provides Zn^2+^ in the form of a water-soluble organic salt and remains covalently bound within the PVA/CS network, enabling controlled sustained release while enhancing crosslinking density and maintaining system uniformity [29].

This study employed alkaline solutions, hydrogen peroxide solutions, and microwave treatment to process balsa wood, removing lignin and hemicellulose from the balsa wood while increasing its porosity to form a porous structure. Using polyvinyl alcohol (PVA), chitosan (CS), and zinc gluconate (ZnG) as primary raw materials, γ-ray irradiation of PVA induced the formation of C–C covalent bonds and enhanced hydrogen bonding interactions, resulting in a dense cross-linked network. CS and PVA form an interpenetrating network through intermolecular hydrogen bonding, while ZnG coordinates with the hydroxyl groups of PVA and the amino groups of chitosan to form a hydrogel. When applied as a wound dressing using balsa wood as the substrate, the BWSM significantly improved the mechanical properties and sustained-release performance of this hydrogel, thereby enhancing its potential applications in wound care. Leveraging the inherent three-dimensional network structure of the hydrogel and the chelation effect between chitosan and zinc ions, precise control over the sustained-release mechanism of ZnG was achieved, endowing the hydrogel with excellent antibacterial properties and prolonged release characteristics. Animal experiments demonstrated that the BWSM/PVA/CS/ZnG hydrogel promotes wound healing in mice and effectively inhibits scar formation, confirming its suitability for biomedical applications such as wound dressings.

## 2. Materials and Methods

### 2.1. Materials

Zinc gluconate (ZnG, 98%), polyvinyl alcohol (PVA, degree of polymerization 1788, alcohol degree 87–89%), sodium hydroxide (NaOH, 99.7%), sodium sulfite (Na_2_SO_3_, 99.7%), and hydrogen peroxide (H_2_O_2_, 30%) were purchased from Shanghai Aladdin Biochemical Technology Co., Ltd. (Shanghai, China). Chitosan (CS, deacetylation 90–91%, viscosity 85 mPa·s) was purchased from Zhejiang Jinke Biotechnology Co., Ltd. (Yuhuan, China). Glacial acetic acid (HAc, 99%) was purchased from Tianjin Zhiyuan Chemical Reagents Co., Ltd. (Tianjin, China). Balsa wood (BW, 0.1 g/cm^3^) was purchased from Guangzhou Qigao Balsa Wood Trading Co., Ltd. (Guangzhou, China). Staphylococcus aureus (*S. aureus*, CMCC 26003) and *Escherichia coli* (*E. coli*, ATCC 8739) were obtained from the Guangdong Provincial Microbial Culture Collection Center (Guangzhou, China).

### 2.2. Treatment of Balsa Wood

Solution treatment of balsa wood: Cut the balsa wood into samples measuring 30 × 30 × 1 mm. Place the balsa wood in a boiling mixture of 2.5 mol/L NaOH and 0.4 mol/L Na_2_SO_3_ aqueous solution and boil continuously for 24 h; the samples treated with NaOH and Na_2_SO_3_ are designated as BWN. Subsequently, immerse the BWN samples in a 2.5 mol/L H_2_O_2_ aqueous solution at 90 °C for 40 min; the resulting samples are named BWS. Finally, rinse the BWS samples four times with 100 °C distilled water to remove the alkaline solution and hydrogen peroxide from the wood.

Microwave treatment of balsa wood: Fully swollen BWS was heated at high microwave power (750 W) for 60 s, inducing extensive cracking in both the surface and interior of the balsa wood and enhancing its porosity, thereby making it suitable as a substrate for hydrogels. The treated samples were designated as BWSM.

### 2.3. Preparation of BWSM/PVA/CS/ZnG Hydrogel

Add PVA powder (5 g) to distilled water (95 g) and continuously stir at 90 °C for 1 h to prepare a 5 wt% PVA solution. Add ZnG (1 g) and CS (2 g) to the solution to form a homogeneous suspension. Then, add acetic acid equivalent to one-third of the mass of CS to the suspension and stir until CS is completely dissolved, yielding a mixed solution. Degas the mixed solution under vacuum for 12 h, transfer it to a centrifuge tube, and pass nitrogen gas through it for gamma-ray irradiation at a dose of 60 kGy with a dose rate of 15–20 Gy/min, resulting in the PVA/CS/ZnG hydrogel. Degas the mixed solution under vacuum for 12 h, transfer it to a centrifuge tube, and pass nitrogen gas through it for gamma-ray irradiation at a dose of 60 kGy with a dose rate of 15–20 Gy/min, resulting in the PVA/CS/ZnG hydrogel. The specific proportions are shown in Table 1.

The BWSM (30 mm × 30 mm) was immersed in the PVA/CS/ZnG mixed solution, followed by vacuum degassing and 48 h of ultrasonic treatment to ensure thorough impregnation of the solution. The impregnated balsa wood was then irradiated with gamma rays at a dose of 60 kGy and a dose rate of 15–20 Gy/min, resulting in the preparation of the BWSM/PVA/CS/ZnG hydrogel dressing (Antibacterial Hydrogel, abbreviated as AH). 

### 2.4. Surface Morphology Observation

The lyophilized samples were cut into 1 cm × 1 cm square specimens. Testing was performed using a scanning electron microscope (SEM, JSM-7500F, Joel, Portland, OR, USA) with an applied voltage of 5 kV. Prior to testing, the samples were fixed on the stage and coated with a gold layer to ensure conductivity; they were then placed on the stage, evacuated for 30 min, and scanned. Mapping analysis was conducted to determine the distribution of nitrogen (N) and zinc (Zn) elements, with evaluation of their distribution depth and uniformity.

### 2.5. Infrared Spectral Analysis

After freezing the hydrogel samples for 12 h, they were subjected to freeze-drying for 48 h to obtain the lyophilized hydrogel samples. Fourier transform infrared spectroscopy (Tensor II FTIR, Bruker, Billerica, MA, USA) was employed for measurement, observation, and analysis of the spectral data. The infrared spectroscopy was performed with a resolution of 4 cm^−1^, a scanning rate of 64 s^−1^, and a scanning range of 500–4000 cm^−1^.

### 2.6. X-Ray Photoelectron Spectroscopy

X-ray Photoelectron Spectroscopy (XPS) was conducted using a Thermo K-Alpha (Thermo Fisher Scientific, Waltham, MA, USA) with a monochromatic Al Kα X-ray source. Prior to sample testing, a lyophilization process was performed.

### 2.7. Differential Scanning Calorimetry Analysis

The thermal properties of balsa wood were characterized using a differential scanning calorimeter (DSC, model 204F1, Netzsch, Bavaria, Germany). The tests were conducted under a nitrogen atmosphere at a heating rate of 10 °C/min, with a temperature range of 30–300 °C.

### 2.8. Thermal Stability Analysis

The thermal stability of balsa wood before and after treatment was determined using a thermogravimetric analyzer (TGA, model 201F1, Netzsch, Bavaria, Germany) at a heating rate of 10 °C/min. The testing temperature range was 30–600 °C.

### 2.9. Quality Loss Analysis

Take a specified mass of balsa wood and subject it to vacuum freeze-drying for 24 h to obtain the lyophilized product, which is then weighed. The lyophilized balsa wood is treated with a solution, followed by drying and weighing of the resulting BWS. The results from three parallel experiments are averaged. The *Mass loss rate* is calculated as follows [30]:

(1) $$Mass\;loss\;rate\left(\%\right)=\frac{W_{0}-W_{1}}{W_{0}}\times 100\%$$

where *W*_0_ and *W*_1_ represent the mass of freeze-dried balsa wood and the mass of freeze-dried balsa wood after solution treatment, respectively.

### 2.10. Swelling Analysis

Soak the lyophilized hydrogel in distilled water for 48 h (at room temperature) until the gel reaches a swelling equilibrium state. Rapidly absorb the surface moisture of the gel with absorbent paper, then weigh it. Repeat the process three times and calculate the average weight. The *Swelling degree* is calculated as follows [31]:

(2) $$Swelling\;degree\left(\%\right)=\frac{W_{s}-W_{d}}{W_{d}}\times 100\%$$

where *W_s_* represents the final hydrogel mass, while *W_d_* denotes the lyophilized hydrogel mass.

### 2.11. Moisture Evaporation Analysis

Take a certain mass of hydrogel and immerse it in distilled water until swelling equilibrium is achieved. Subsequently, place the sample in an oven (temperature 50 °C, humidity 50%) and measure the sample mass every hour until a constant weight is reached. Repeat the experiment three times in parallel, and calculate the average value. The moisture evaporation rate is calculated as follows [32]:

(3) $$Water\;evaporation\;rate\left(\%\right)=\frac{W_{1}-W_{2}}{W_{1}-W_{3}}\times 100\%$$

where *W*_1_, *W*_2_, and *W*_3_ represent the initial mass, measured mass, and final weight of the hydrogel, respectively.

### 2.12. Mechanical Performance Analysis

The mechanical properties of balsa wood were tested using a mechanical testing machine (CMT 0102, Ssans, Shanghai, China). Samples of balsa wood, both before and after treatment, were cut to dimensions of 30 × 10 × 1 mm. During the mechanical tests, the gauge length was 20 mm, and the tensile rate was 2 mm/min. Each sample was tested three times in parallel, and the average value was recorded.

### 2.13. Specific Surface Area Analysis

The specific surface area of the adsorbent was determined using a fully automated gas adsorption analyzer (BET, ASAP2460, Micromeritics, Norcross, GA, USA). First, the adsorbent underwent a 3 h vacuum pretreatment at 120 °C. Subsequently, N_2_ adsorption–desorption tests were conducted in liquid nitrogen, and the specific surface area was calculated using the Brunauer–Emmett–Teller model.

### 2.14. In Vitro Sustained Release of Zinc Gluconate

Place 1.3 g of hydrogel in 50 mL of PBS saline buffer (37 °C, pH 7.4) and maintain mechanical stirring. At regular intervals (3, 6, 9, 12, 24, 48 and 72 h), aspirate 2 mL of the buffer solution and add an equal volume of fresh PBS buffer to maintain constant total buffer volume. The release amount of zinc ions was determined using atomic absorption spectroscopy (AAS, NovAA400P, Analytic Jena, Jena, Germany).

### 2.15. Antibacterial Performance Analysis

Antibacterial activity against Staphylococcus aureus and *Escherichia coli* was determined. Hydrogel samples were prepared as square specimens with sides approximately 1 cm in length for the antibacterial assay. After ultraviolet irradiation for 15 min to sterilize the samples, they were cultured for 12, 24, 48 and 72 h in bacteria-coated agar medium at a constant temperature of 37 °C. The size of the antibacterial zone surrounding the hydrogel on the agar plate reflected its antibacterial activity, and the radius of the bacterial growth inhibition zone was measured using Dig simulator software.

### 2.16. Cytotoxicity Testing

A 100  μL mouse primary fibroblast suspension was placed in wells of a 96-well plate. The plates were precultured in an incubator for 30–45 min (37 °C, 5% CO_2_). After UV light disinfection, the samples were placed in the culture plate. After incubation, the medium was replace with fresh culture medium preheated to 37 °C and incubate in the dark for 30 min at 37 °C to ensure that the cell lactonase fully hydrolyzes Calcein AM and generates Calcein with green fluorescence. Remove the culture medium, wash 2–3 times with PBS, and then add serum-free cell culture medium for observation under a fluorescence microscope or detection using a fluorescence enzyme-linked immunosorbent assay reader. The solution was measured at the absorption peak of 450  nm with a microplate reader. The mean and SD were used as the experimental results. The relative activity of the cells in the experimental and blank groups was compared. *Cell viability* was calculated as follows:

(4) $$Cell\;viability\;=\;\frac{{OD}_{ex}-{OD}_{b}}{{OD}_{n}-{OD}_{b}}\;\times \;100\%$$

where ${OD}_{ex}$ was the optical density of the experimental group, ${OD}_{b}$ was the optical density of the blank background group and ${OD}_{n}$ was the optical density of blank control group. The average of three measurements was taken as the results of cell viability.

### 2.17. Animal Experiments

Sixteen high-standard hygienic mice were used, all of which were acclimated to the environment for one week prior to surgery. The 16 mice were randomly divided into four groups (n = 4): the control group (without dressing), AH-1 group, AH-2 group, and AH-3 group, with four mice in each group labeled accordingly. Under sterile conditions, all mice were anesthetized with isoflurane, and their backs were scraped and cleaned with 70% ethanol. A full-thickness wound (8 mm in length and 8 mm in width) was then created, and the dressing was sterilized under ultraviolet light for 15 min. The sterilized dressing was sutured onto the wounds of each group using sterile surgical sutures (with the wood surface facing outward and the gel surface adhering to the wound). The exposed wounds served as controls. The dressings were changed every three days, and the wound area was measured using a vernier caliper, with photographs taken on postoperative days 1, 5, 10, and 15 to assess and document the wound appearance. All animal experiments were approved by the Medical Ethics Committee of Harbin Medical University (Harbin, China) and were carried out in accordance with the National Institutes of Health Standards for Laboratory Animal Research (National Institutes of Health (NIH), Bethesda, MD, USA), Licence: SYDW2025-081 (10 June 2025). Mice were purchased from Beijing Vital River Laboratory Animal Technology Co., Ltd. (Beijing, China).

### 2.18. Statistical Analysis

The data in the graphs and text were presented as mean ± SD (standard deviation), and statistical comparisons were made using Tukey’s test. *p*-values < 0.05 were considered to be statistically significant. n.s.: no significance; * *p* ≤ 0.05; ** *p* ≤ 0.01; *** *p* ≤ 0.001.

## 3. Results and Discussion

### 3.1. Structural Analysis of the Processed Balsa Wood

**Figure 1:**
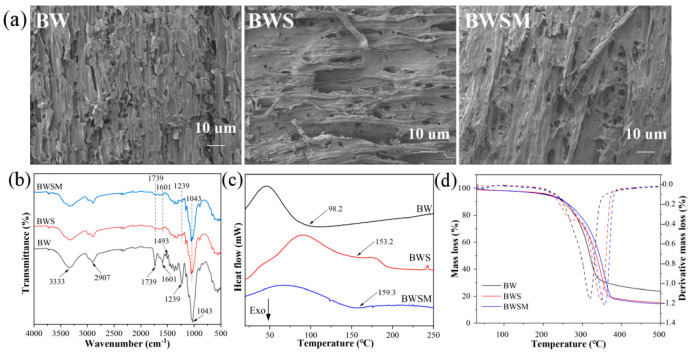
(**a**) Surface morphology of balsa wood before and after treatment; (**b**) Infrared image of balsa wood before and after treatment; (**c**) DSC curve of balsa wood before and after treatment; (**d**) TGA curve of balsa wood before and after treatment.

As shown in Figure 1a, the BW cross-section exhibits a well-defined structure with intact cellular architecture and no prominent macropores. After treatment with NaOH/Na_2_SO_3_ and H_2_O_2_, BWS was obtained, demonstrating partial removal of lignin and hemicellulose along with moderate damage to the cell walls and intercellular layers. The radial cross-section reveals numerous uniformly distributed pores with significantly increased pore size and porosity, though the wood structure appears disordered and the surface microstructure shows some degradation, indicating structural alteration and reduced mechanical strength of BW. Further microwave treatment yielded BWSM, where thin-walled cells are destroyed and the intercellular layer is torn, reflecting further deterioration of BW’s internal structure. The radial cross-section becomes more irregular, with evident cell wall delamination, reflecting enhanced porosity in the treated BW. This softer structure improves material permeability and facilitates subsequent hydrogel loading.

In Figure 1b, the absorption peak at 3333 cm^−1^ in BW corresponds to the O–H stretching vibration, while the peak at 2907 cm^−1^ represents the C–H stretching vibration. The infrared absorption peak at 1739 cm^−1^ originates from the acetyl group in hemicellulose, the characteristic absorption peak at 1601 cm^−1^ corresponds to the C=O bond, the peak at 1493 cm^−1^ reflects the stretching vibrations of aromatic rings in lignin, the peak at 1239 cm^−1^ indicates vibrations of the fatty acid group, and the peak at 1043 cm^−1^ represents in-plane deformation of the C–H (aromatic) bonds in lignin. Compared to BW, both BWS and BWSM exhibit significant reductions in the characteristic absorption peaks at 1739 cm^−1^, 1601 cm^−1^, 1239 cm^−1^, and 1043 cm^−1^, demonstrating that the solution treatment removed most of the lignin and hemicellulose from balsa wood. Additionally, no new characteristic absorption peaks emerged in BWSM, indicating that the microwave treatment did not alter the chemical structure of balsa wood and preserved its cellulose-based macromolecular framework [33,34,35].

As shown in Figure 1c, the exothermic peak of BW at 98.2 °C is attributed to the crystallization of cellulose in the wood, whereas in BWS, this peak shifts significantly toward higher temperatures to 153.2 °C. This shift occurs because the solution treatment removes most of the lignin and hemicellulose from balsa wood, thereby increasing its cellulose content and enhancing thermal stability. Further microwave treatment yields BWSM, whose exothermic peak continues to shift toward higher temperatures to 159.3 °C, demonstrating that microwave treatment further improves the thermal stability of balsa wood.

As shown in Figure 1d and Table 2, the weight loss profile of BWS is divided into three stages. The first stage spans 0–173 °C, with a mass loss rate of approximately 3.6%, primarily attributed to the loss of free water and crystallization water. The second stage (173–337 °C) exhibits more pronounced mass loss, reaching a rate of 70.9%, with the maximum thermal weight loss rate occurring at 318.7 °C; this stage is mainly characterized by the pyrolysis of cellulose and hemicellulose. The third stage (337–500 °C) involves weight loss due to lignin pyrolysis. The maximum thermal weight loss range of BWS shifts significantly to 301–362 °C, with a mass loss rate of 79.2%—higher than that of balsa wood. This higher rate results from the removal of substantial amounts of lignin and hemicellulose, leading to an increased specific gravity and consequently a greater weight loss. The maximum thermal weight loss rate for BWS rises to 346.9 °C, indicating that the solution treatment enhances the thermal stability of balsa wood. The maximum mass loss range of BWSM shifts to 309–371 °C, with a mass loss rate of 82.0% in this interval. The maximum thermal weight loss rate occurs at 356.7 °C, indicating that microwave treatment enhances the thermal stability of balsa wood. This is attributed to the degradation of cellulose and hemicellulose components in the wood during microwave treatment. However, due to the lower polymerization degree and higher chemical activity of hemicellulose, coupled with cellulose’s superior crystallinity, microwave degradation primarily occurs in hemicellulose [36,37].

### 3.2. Performance Analysis of Balsa Wood After Treatment

The mass loss of the treated balsa wood was analyzed. As shown in Table 3, the mass loss rate of BWN was 33.57 ± 2.01%. The treatment of balsa wood with NaOH and Na_2_SO_3_ solutions primarily involves selective hydrolysis and removal of hemicellulose and lignin. NaOH promotes the hydrolysis of acetyl ester bonds in hemicellulose side chains, triggering alkaline hydrolysis of main-chain glycosidic bonds and terminal group detachment; in the presence of Na_2_SO_3_, phenolic units of lignin are activated, undergoing a coupled reaction of nucleophilic attack and *α*-aryl ether bond cleavage, yielding more water-soluble fragments that achieve partial delignification. Cellulose remains relatively stable; consequently, the treated material (BWN) transforms into a porous network with a continuous cellulose skeleton, exhibiting significantly reduced hemicellulose content and partial lignin removal, thereby enhancing wood flexibility and ability to bend under high curvature. While retaining essential structural strength due to the preserved cellulose framework, this material proves particularly suitable as a lignocellulosic substrate for hydrogel loading [38,39].

**Figure 2:**
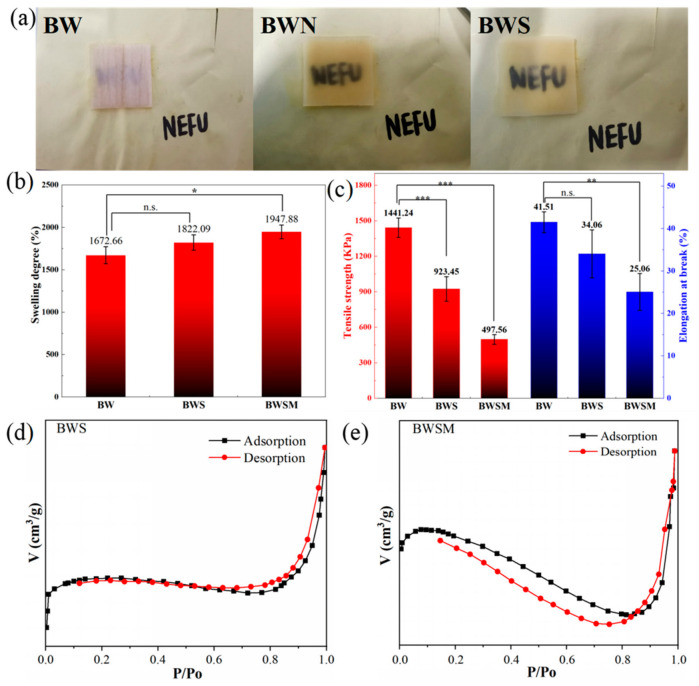
(**a**) Physical appearance of balsa wood before and after treatment; (**b**) Swelling degree of balsa wood before and after treatment; (**c**) Mechanical properties of balsa wood before and after treatment; (**d**) Adsorption–desorption isotherms of BWS; (**e**) Adsorption–desorption isotherms of BWSM (in the bar chart: n.s.: no significance; * *p* ≤ 0.05; ** *p* ≤ 0.01; *** *p* ≤ 0.001 (n ≥ 3)).

Figure 2a shows the color and transparency of balsa wood before and after treatment. Compared with BW, BWN treated with NaOH/Na_2_SO_3_ exhibits significantly increased transparency and a lighter color, primarily because the color of balsa wood originates mainly from numerous chromophoric and auxochromophoric groups in lignin, as well as extracts such as tannins and pigments; the NaOH/Na_2_SO_3_ treatment removes substantial amounts of lignin and hemicellulose from balsa wood [40,41]. After rinsing BWN with H_2_O_2_ for 40 min, BWSM was obtained. The wood chips of BWSM exhibited further lightening in color, but their transparency showed little improvement. This is because H_2_O_2_ primarily removes chromophores from lignin and pigments from the extracts, while only a small portion of lignin is eliminated. Wood treated with NaOH/Na_2_SO_3_ followed by H_2_O_2_ treatment demonstrated significantly enhanced transparency and lighter coloration. These results indicate that BW, as a hydrogel substrate, undergoes solution treatment that improves its inherent transparency, enabling clearer visualization of wounds and facilitating dynamic observation of wound healing progress.

Swelling capacity is a critical indicator for evaluating wound dressings. To determine whether BWSM can serve as a suitable dressing substrate, swelling tests were conducted. As shown in Figure 2b, solution treatment increased the swelling degree of balsa wood from 1672% to 1822%, while microwave treatment elevated it further from 1822% to 1947%. These results demonstrate that both solution and microwave treatments enhance the wood’s swelling capacity. This improvement stems from the treated balsa wood exhibiting higher porosity and a more developed pore structure, which enhances water transport within the wood and facilitates smoother fluid flow through the vascular system. Additionally, cell wall rupture further accelerates fluid penetration within the wood, thereby improving its swelling performance [42].

As shown in Figure 2c, tensile strength tests were conducted on balsa wood before and after treatment. Compared to BW, the tensile strength and elongation at break both decreased in BWS after removal of most lignin and hemicellulose: the tensile strength dropped from 1441.2 kPa to 923.5 kPa, and the elongation at break decreased from 41.5% to 34.1%. This is primarily because the microfilaments of cellulose in balsa wood provide mechanical support for the cell walls, while the hemicellulose and lignin matrix or crust fills the cellulose framework. Solution treatment removed substantial amounts of hemicellulose and lignin, along with a portion of cellulose, thereby compromising the wood’s mechanical properties. Microwave treatment further reduced the tensile strength and elongation at break of BWSM, with the tensile strength decreasing from 923.5 kPa to 497.6 kPa and the elongation at break decreasing from 34.1% to 25.1%. This occurs because steam pressure generated during microwave treatment induces more internal cracks in the balsa wood, leading to diminished mechanical performance.

To further investigate the effects of microwave treatment on the structure, specific surface area, and pore size of balsa wood, specific surface area analyses were conducted on both BWS and BWSM. As shown in Table 4, the BET specific surface area of BWS is 8.0 m^2^/g, while that of BWSM is slightly lower at 5.8 m^2^/g. The reduction in the specific surface area of balsa wood after microwave treatment is attributed to the following processes: During microwave treatment, partial hydrolysis of hemicellulose generates CH_3_COOH; cellulose dehydration leads to the formation of β–O bonds; and lignin undergoes esterification reactions, resulting in a decrease in hydroxyl groups and reduced adsorption capacity. Additionally, the internal pore structure of the treated wood becomes more complex, containing pores of varying sizes. While microwave treatment increases intercellular fissures, it reduces the number of micropores (those smaller than 2 nm). Since micropores exhibit stronger physical adsorption than the external surface, this contributes to the decrease in the specific surface area of microwave-treated balsa wood [43,44]. The BET adsorption pore size of BWS is 5.8 nm, while that of BWSM is 6.3 nm. The larger pore size in BWSM compared to BWS results from the further disruption of the wood’s internal structure and increased porosity following microwave treatment, leading to enlarged adsorption pores.

As shown in Figure 2d,e, the adsorption and desorption isotherms of both BWS and BWSM exhibit Type II isotherms, consistent with the characteristics of BW multilayer adsorption. In Figure 2d, the adsorption and desorption curves close at low pressure without any hysteresis loop, indicating a relatively uniform shape and size of BWS. In contrast, in Figure 2e, the curves fail to close at low pressure and show hysteresis, suggesting non-uniform pore morphology and size in BWSM—a phenomenon attributed to microwave treatment-induced fractures between wood cell walls.

### 3.3. Structural Analysis of the BWSM/PVA/CS/ZnG Hydrogel

**Figure 3:**
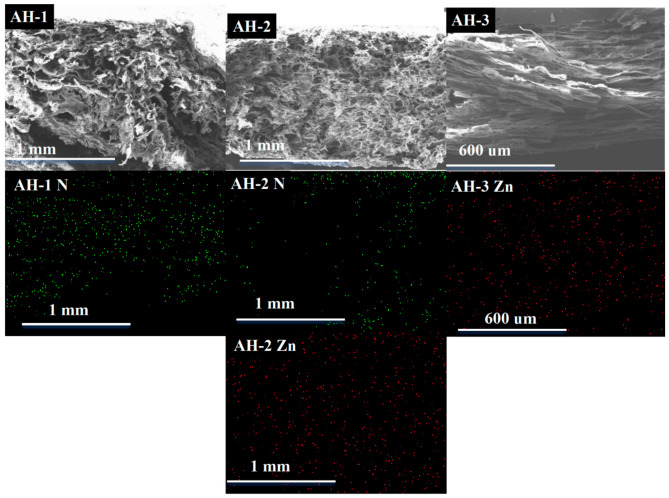
Analysis of the surface morphology of AH hydrogel.

The surface and cross-sectional morphology of the hydrogel dressing were investigated using SEM, and elemental analyses of nitrogen (N) and zinc (Zn) were performed on its cross-section. As shown in Figure 3, the dressing with BWSM as the substrate exhibits a loose, porous surface with uniformly distributed hydrogel. The pores of both the hydrogel and the balsa wood not only provide pathways for the passage of water vapor, oxygen, and wound exudate but also create a favorable environment for cell adhesion and growth, enabling rapid delivery of CS and ZnG to the wound surface. Additionally, the fundamental structure of the balsa wood is preserved, maintaining the dressing’s mechanical strength. Compared to AH-3 without CS, AH-1 and AH-2, which contain CS, demonstrate higher porosity due to the high-dose degradation of CS. X-ray energy dispersive spectroscopy analysis of the hydrogel dressing cross-section revealed uniform distributions of N and Zn across the wood surface, indicating thorough impregnation of the hydrogel within the wood. However, AH-2 exhibited slightly inferior impregnation performance compared to AH-1 and AH-3. This is attributed to the chelation effect between Zn^2+^ ions and the –NH_2_ groups on CS when ZnG and CS are co-present in solution, resulting in significantly higher viscosity of the CS/PVA/ZnG mixture compared to the PVA/CS or PVA/ZnG mixtures, thereby hindering effective impregnation into the wood. In conclusion, the fundamental structure of BWSM remains intact, and the hydrogel is fully loaded within the BWSM matrix.

**Figure 4:**
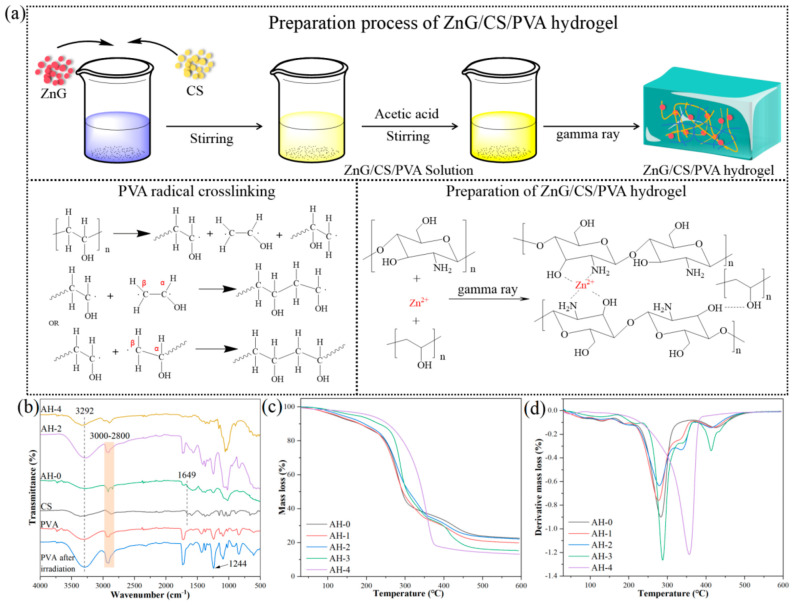
(**a**) Hydrogel preparation process and related chemical equations; (**b**) Infrared analysis image of the hydrogel; (**c**) TGA curve of the hydrogel; (**d**) DTG curve of the hydrogel.

As shown in Figure 4a,b, the FTIR spectrum of irradiated PVA hydrogel showed no new characteristic peaks or disappearance of existing peaks, indicating that γ-ray irradiation induced cross-linking without introducing new functional groups. Notably, the enhanced O–H stretching vibration at 3292 cm^−1^ and the intensified absorption peak near 1244 cm^−1^ (attributable to C–O stretching and O–H bending modes) demonstrate strengthened hydrogen bonding interactions during irradiation. These enhanced physical interactions synergize with radiation-induced C–C covalent bond formation, promoting densification of the PVA network structure [45,46,47]. Upon incorporation of chitosan (CS) into the PVA matrix (AH-0), the broad O–H stretching peaks at 3292 cm^−1^ significantly broadened due to enhanced intermolecular hydrogen bonding, while the C–H stretching peaks (3000–2800 cm^−1^) remained consistent with those of native PVA. No new absorption peaks associated with covalent bonds (e.g., imine groups C=N) emerged, confirming that PVA and CS coexist in a physically interconnected network rather than through chemical copolymerization [48,49]. Addition of ZnG attenuated the characteristic amide I peak at 1649 cm^−1^ in chitosan, attributed to coordination between Zn^2+^ ions and electronegative atoms (N/O) in the polymer matrix, disrupting the original carbonyl vibration modes. Furthermore, the FTIR spectrum of the hydrogel loaded with Balsa wood (AH-2) showed no new absorption peaks compared to that of the hydrogel dressing (AH-0), indicating that the loading process resulted solely in a pure physical composite without altering the physicochemical interactions among PVA, CS, and ZnG [50].

As shown in Figure 4c,d and Table 4, AH-0 is a PVA/CS/ZnG hydrogel that exhibits the most significant mass loss between 252 and 308 °C, with a mass loss rate of 55.9%. During this temperature range, both CS and PVA begin to decompose, with the maximum thermal weight loss rate occurring at 283.0 °C. After loading the hydrogel onto BWSM, AH-2 demonstrates no notable change in thermal stability; its most severe mass loss occurs between 250 and 308 °C, and the maximum thermal decomposition rate is observed at 279.4—almost identical to that of the pure hydrogel. Additionally, a new thermal decomposition region emerges between 341 and 414 °C, corresponding to AH-4′s maximum decomposition range (309–371 °C), indicating no chemical changes during the loading of the hydrogel onto balsa wood.

As shown in Figure 4c,d and Table 5, the maximum thermal decomposition rates of the three hydrogel dressings—AH-1, AH-2, and AH-3—are largely comparable. AH-1 and AH-2 exhibit three distinct thermal decomposition zones: the first zone occurs before 250 °C, with a mass loss of approximately 8%, primarily due to the removal of free and bound water from the material. The maximum thermal weight loss for all three dressings occurs around 280 °C, corresponding to the thermal decomposition stages of CS and PVA, while a distinct peak appears at approximately 340 °C, matching the thermal decomposition temperature of BWSM. The AH-3 sample exhibits a distinct thermal decomposition peak at 414.6 °C, consistent with the decomposition temperature of ZnG; in contrast, the AH-2 sample containing ZnG shows no pronounced decomposition peak—its behavior resembling that of the ZnG-free AH-1. This phenomenon likely stems from chelation between the amino groups in AH-2 and ZnG, which may mask or shift the decomposition peak to other temperature regions, thereby eliminating the peak observed at 414.6 °C.

**Figure 5:**
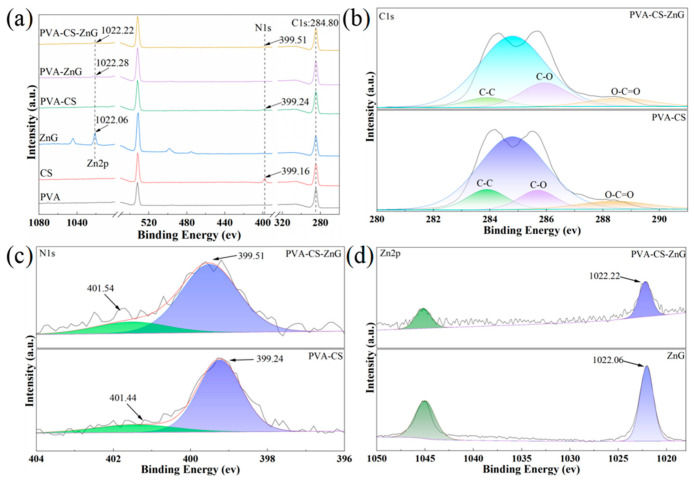
XPS survey of (**a**) polyvinyl alcohol/chitosan/zinc gluconate and high-resolution XPS of (**b**) C1s, (**c**) N1s, and (**d**) Zn2p.

The chemical state and surface elemental composition of the polyvinyl alcohol/chitosan/zinc gluconate hydrogel were characterized using X-ray photoelectron spectroscopy (XPS). As shown in Figure 5a,b, peaks at 283.79 eV, 285.97 eV, and 288.65 eV in the C1s spectrum correspond to C–C, C–O, and O–C=O bonds, respectively. The prominent C–O and O–C=O signals at 285.97 eV and 288.65 eV are consistent with the introduction of carboxyl groups from ZnG. High-resolution N1s spectroscopy revealed key interactions: as illustrated in Figure 5c, PVA–CS exhibits a new characteristic peak at 401.44 eV compared to pure CS (399.16 eV), attributed to protonation of –NH_2_ groups into –NH_3_^+^ by residual acetic acid; this peak shifts further to 401.54 eV upon addition of ZnG, indicating reduced electron density around nitrogen atoms and suggesting Zn–N coordination or electrostatic interactions involving Zn^2+^. This conclusion is supported by Figure 5d, where the Zn2p peak in PVA–CS–ZnG (1022.22 eV) shifts +0.16 eV relative to that in pure ZnG (1022.06 eV). The simultaneous positive shifts in both N1s energy and Zn2p binding energy provide strong evidence for coordination between Zn^2+^ and amino/hydroxyl groups within the PVA–CS network [51,52].

### 3.4. Performance Analysis of the BWSM/PVA/CS/ZnG Hydrogel

**Figure 6:**
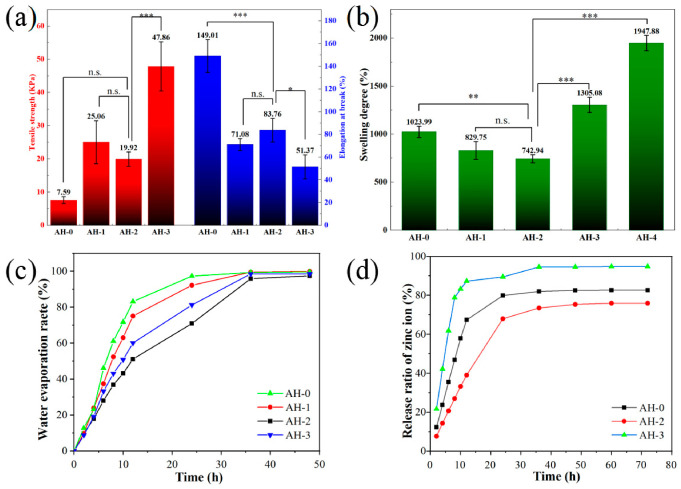
(**a**) Mechanical properties of the AH hydrogel; (**b**) Swelling curve of the AH hydrogel; (**c**) Water evaporation rate curve of the AH hydrogel; (**d**) In vitro sustained-release profile of ZnG (in the bar chart: n.s.: no significance; * *p* ≤ 0.05; ** *p* ≤ 0.01; *** *p* ≤ 0.001 (n ≥ 3)).

As shown in Figure 6a, the addition of BWSM increased the hydrogel’s tensile strength to 265% of its original value, while the elongation at break decreased from 149.0% to 83.8%. In comparison with AH-1, AH-2, and AH-3, it was observed that the incorporation of chitosan and gluconate reduced the hydrogel’s tensile strength. This is attributed to the fact that the hydrogel’s irradiation-crosslinked backbone consists of PVA; the addition of CS and ZnG affects the crosslinking degree of PVA, thereby compromising the hydrogel’s mechanical properties.

As shown in Figure 6b, swelling analyses were conducted on three dressings with different compositions, hydrogels, and dressing substrates. AH-4 exhibited the highest swelling degree, reaching 1947%. This is attributed to the inherent pore structure of balsa wood, whose weight is only one-third that of an equivalent volume of water. Additionally, solution treatment and microwave treatment endowed BWSM with a softer and more porous structure, significantly enhancing AH-4′s swelling capacity. Compared to the pure hydrogel AH-0, the swelling degree of AH-2 (the dressing loaded with BWSM) decreased from 1024% to 742%. This reduction occurs because the hydrogel fills the pores of the balsa wood and is entrapped within the wood matrix, thereby suppressing the permeability and swelling properties of both the hydrogel and the wood. In contrast to AH-1 and AH-3, AH-2 demonstrated the lowest swelling degree. The high swelling degree of AH-3 (1305%) stems from the abundant hydrophilic groups present in PVA and ZnG, which confer enhanced swelling performance. However, in AH-2 supplemented with CS, the amino groups on CS chelate with ZnG, occupying some of its hydrophilic groups. The higher swelling degree of AH-1 than AH-2 results from the irradiation-induced degradation of chitosan, which increases the porosity of the PVA gel network; however, the addition of ZnG inhibits this degradation process. Consequently, AH-2 exhibited the lowest swelling degree at 742%.

Water evaporation rate is one of the key criteria for evaluating dressings, as a moist environment facilitates wound healing and reduces scar formation. As shown in Figure 6c, the hydrogel dressing loaded with BWSM exhibits a lower water evaporation rate compared to pure hydrogel. This is attributed to the hydrogel being embedded within BWSM, where the inherent thermal and moisture-retaining properties of balsa wood enhance moisture retention. AH-2 and AH-3 demonstrate lower water evaporation rates than AH-1 due to the high-irradiation-induced degradation of chitosan, which increases the hydrogel’s porosity; additionally, Zn^2+^ in AH-3 binds to PVA chains, restricting their mobility and thereby reducing water loss. AH-2 shows the lowest water evaporation rate because the simultaneous presence of CS and ZnG induces chelation between chitosan and zinc ions, increasing cross-linking density and forming a denser internal network that resists water loss. Consequently, AH-2 with the lowest water evaporation rate is more suitable for use as a wound dressing.

As shown in Figure 6d, all three hydrogel dressings contained 1% ZnG. All three groups exhibited rapid release within the first 10 h: AH-0 released 58.0%, AH-2 released 33.2%, and AH-3 released a remarkable 83.3%. By 24 h, the release rates of zinc ions exceeded 60% for all dressings—AH-0 reached 79.9%, AH-2 68.0%, and AH-3 89.5%. After 24 h, AH-3′s release stabilized, while AH-0 and AH-2 continued to release slowly, reaching 82.6% and 75.4%, respectively, by 48 h. The results demonstrate that the addition of CS modulated the ZnG release rate, reducing its rate while extending the sustained release duration. This effect stems from the coordination between the amino groups on CS and ZnG, enabling prolonged and stable release. The presence of balsa wood also inhibited the ZnG release rate, as the wood suppressed the hydrogel’s permeability and swelling capacity, thereby reducing swelling and consequently suppressing the release rate over time.

### 3.5. Antibacterial Activity, Cytotoxicity and Animal Experiments of the BWSM/PVA/CS/ZnG Hydrogel

**Figure 7:**
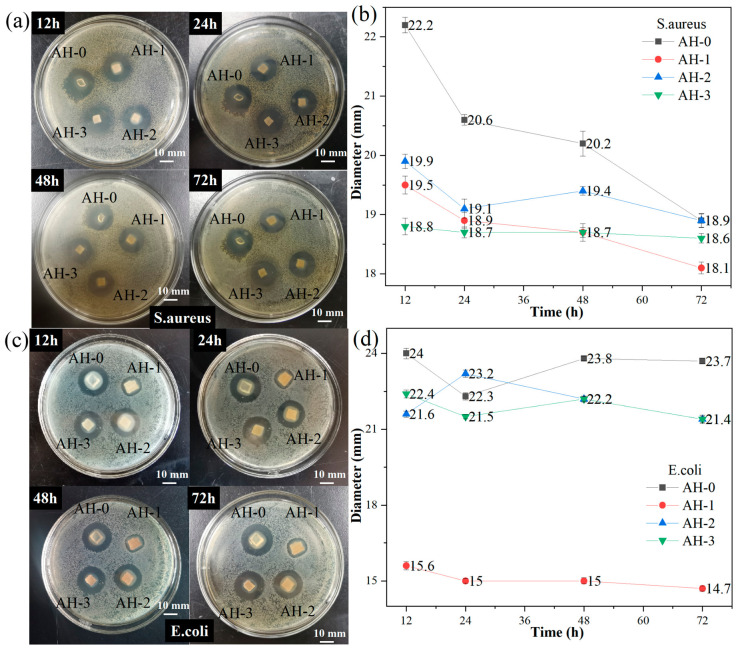
(**a**) Actual image of AH hydrogel inhibiting Staphylococcus aureus; (**b**) Curve graph of AH hydrogel inhibiting Staphylococcus aureus; (**c**) Actual image of AH hydrogel inhibiting *Escherichia coli*; (**d**) Curve graph of AH hydrogel inhibiting *Escherichia coli*.

Figure 7a demonstrates the antibacterial efficacy of hydrogel dressings against Staphylococcus aureus, while Figure 7b shows the diameters of the antibacterial zones. Clearly, all experimental groups exhibited significant antibacterial activity against *S. aureus*, with the two active components responsible for this effect being chitosan (CS) and zinc particles (ZnG), respectively. Within 12 h, the antibacterial zone diameters of AH-1 (containing only CS) were nearly identical to those of AH-3 (containing only ZnG), although the diameter of the CS-based dressing was slightly larger than that of the ZnG-based dressing. After 24 h, the antibacterial zone diameter of AH-1 gradually decreased, whereas that of AH-3 remained stable, indicating that ZnG demonstrated superior long-term antibacterial efficacy compared to CS. Notably, AH-2 (containing both active components) consistently exhibited a larger antibacterial zone diameter than AH-1 and AH-3 over 72 h, attributed to the synergistic interaction between ZnG and CS, where the latter further enhances antibacterial effects by disrupting bacterial cell membranes. AH-0 and AH-2 exhibit distinctly different antibacterial time-curves, which stem from the carrier’s controlled sustained release of ZnG. In AH-0, ZnG is relatively uniformly distributed within the hydrogel; upon contact with moist agar, the gel rapidly releases a large amount of Zn^2+^ ions, resulting in the largest initial antibacterial zone, which subsequently diminishes significantly within 72 h. In contrast, AH-2 primarily encapsulates ZnG inside or on the surface of BWSM, establishing a drug delivery pattern with longer duration but diffusion-dependent characteristics. Consequently, AH-2 exhibits a smaller initial antibacterial zone that remains more stable, whereas AH-0 demonstrates a faster decline in its antibacterial efficacy [53,54]. Figure 7c shows the antibacterial efficacy of hydrogel dressings against *Escherichia coli*, while Figure 7d displays their antibacterial zone diameters. All dressings demonstrated effective antibacterial properties against *E. coli*. However, AH-1 (without ZnG) exhibited an antibacterial zone diameter of approximately 15 mm—only 70% of AH-3′s efficacy—while AH-2 showed minimal difference from AH-3, confirming that ZnG is the primary antibacterial component in these dressings. The efficacy of wound dressings varies between Gram-negative and Gram-positive bacteria, with the former exhibiting superior performance due to the following reasons: For *S. aureus* (Gram-positive bacterium), due to its absence of an outer membrane, CS reacts with the negatively charged cell membrane, leading to the loss of intracellular proteins and thereby exerting an antibacterial effect. *E. coli* (Gram-negative bacterium) is encased by a robust outer membrane that exhibits structural stability. The Zn^2+^ ions released by the hydrogel competitively displace divalent cations from the membrane, thereby compromising its integrity; within the bacterial cell, they induce metal homeostasis disruption, indirectly deplete sulfhydryl groups to enhance oxidation, and impair bacterial activity, ultimately exerting a synergistic effect with the cations in CS—this explains the observed broader spectrum of inhibitory activity against *E. coli* [55,56].

**Figure 8:**
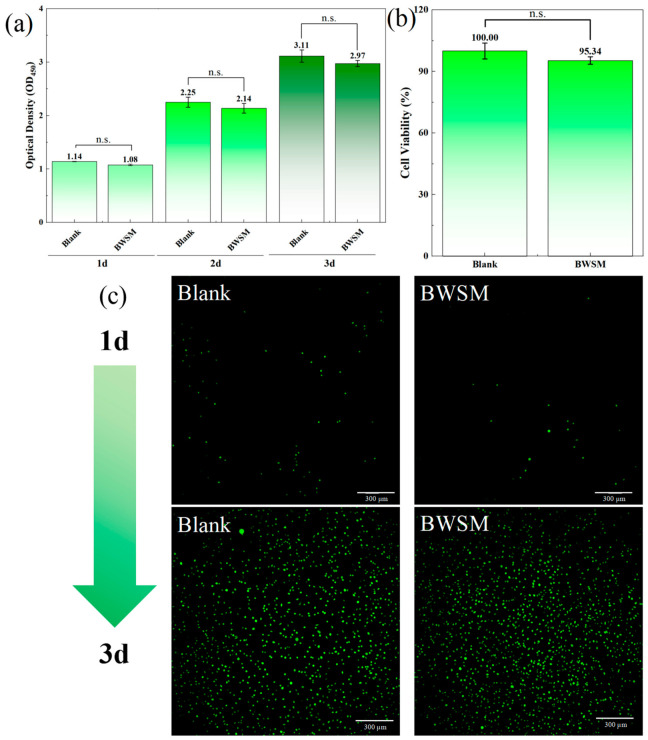
Results of cytotoxicity testing for BWSM: (**a**) Optical density values of BWSM; (**b**) Cell survival rates of BWSM; (**c**) Fluorescence images obtained from live cell counting assays (in the bar chart: n.s.: no significance; (n ≥ 3)).

Our previous studies demonstrated that the cell survival rates were 108.25% for AH-0 (PVA–CS–ZnG ratio 5:2:1), 104.33% for CS-3 (PVA–CS–ZnG ratio 5:3:1), and 102.91% for Zn-3 (PVA–CS–ZnG ratio 5:2:3) [57]. As shown in Figure 8, the cell survival rates in the newly added BWSM experimental group were above 95%, indicating excellent biocompatibility. The relatively lower cell survival rate of BWSM (95.34%) may be attributed to the inhibitory effect of residual lignin/hemicellulose or the spatial barriers imposed by its rough microstructure on cell adhesion. In contrast, the AH-0 group exhibited the highest cell viability (108.25%), demonstrating that the PVA–CS–ZnG matrix effectively supports cellular metabolism without inducing cytotoxicity. For composite dressings, excessive addition of CS or ZnG resulted in a slight decrease in cell survival rates (CS-3: 104.33%; Zn-3: 102.91%). This minor decline is likely due to increased surface cation density and altered swelling degree/morphology of the scaffold caused by excess CS, thereby affecting interfacial interactions between cells and the material; additionally, elevated Zn^2+^ ion release at high concentrations may disrupt the cellular microenvironment or induce mild oxidative stress. Overall, the PVA–CS–ZnG hydrogel combined with a modified wood matrix exhibits superior biosafety and serves as an ideal candidate material for wound dressing applications [58,59,60].

**Figure 9:**
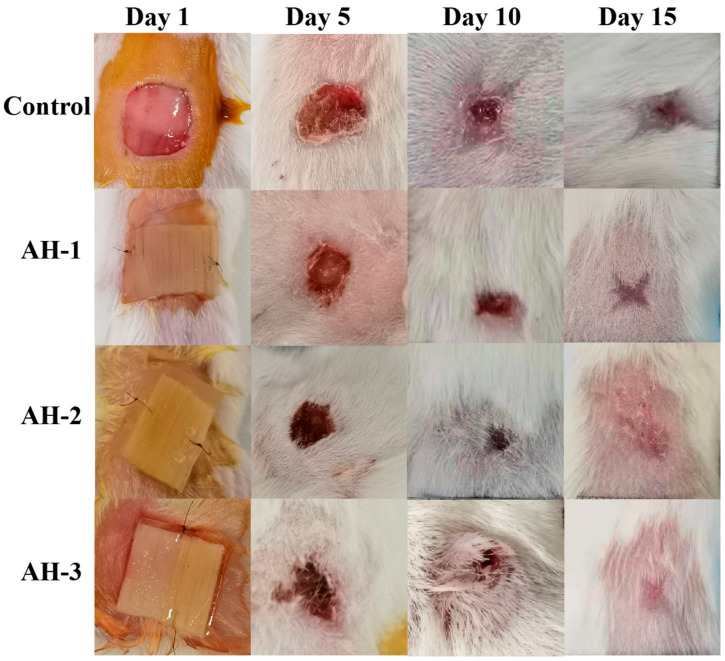
Animal experiment results of AH hydrogel.

Since the skin serves as a critical barrier protecting the host from external microorganisms and preventing dehydration, a full-thickness skin defect repair experiment was conducted in mice to evaluate the wound healing efficacy of the BWSM/PVA/CS/ZnG hydrogel dressing. Wound healing progress was observed on days 1, 5, 10, and 15. The experimental results are shown in Figure 9, demonstrating that all three dressings exhibited superior healing outcomes compared to the control group without dressing application. On day 5, the wound sizes were as follows: control (9 mm × 7.3 mm), AH-1 (5.5 mm × 6.3 mm), AH-2 (4.7 mm × 4.8 mm), and AH-3 (4.4 mm × 8.0 mm). These results indicate that all three dressings possess excellent antibacterial properties, with AH-2 demonstrating the most effective wound healing. By day 10, the wound sizes were: control (3.2 mm × 3.9 mm), AH-1 (3.0 mm × 5.2 mm), AH-2 (2.9 mm × 3.0 mm), and AH-3 (1.9 mm × 2.3 mm). While the control group exhibited a reduced wound area, the wound remained deeper; in contrast, the other three groups showed significantly smaller and shallower wounds. This confirms that CS and ZnG exhibit superior properties for deep wound healing. On day 15 of the experiment, the wound size in the control group was 2.4 mm × 2.8 mm, while the other three dressing-treated groups had fully healed—particularly AH-2, which showed no significant scarring. This demonstrates that the BWSM/PVA/CS/ZnG hydrogel dressing not only exhibits excellent antibacterial and healing properties but also effectively prevents scar formation in wounds.

## 4. Conclusions

This study employed BWSM as the hydrogel substrate, with PVA, CS, and ZnG as the primary raw materials, and utilized gamma-ray irradiation to prepare the BWSM/PVA/CS/ZnG hydrogel, which exhibits superior antibacterial properties and sustained release performance. Balsa wood treated with alkaline solution, hydrogen peroxide, or microwave treatment demonstrated enhanced transparency, increased porosity, and preserved mechanical strength, while its thermal stability and swelling capacity were also improved. The gamma-ray-irradiated BWSM/PVA/CS/ZnG hydrogel wound dressing showed, through sustained-release and antibacterial tests, that the PVA/CS/ZnG hydrogel loaded onto balsa wood achieved prolonged drug release and antibacterial effects. Animal experiments confirmed that the BWSM/PVA/CS/ZnG hydrogel promotes wound healing in mice and effectively prevents scar formation. In summary, the BWSM/PVA/CS/ZnG hydrogel possesses durable antibacterial properties, high mechanical strength, and wound-healing capabilities, making it suitable for applications in biomedical materials such as wound dressings.

Although balsa wood effectively compensates for the inherent low modulus and poor dimensional stability of PVA/CS/ZnG hydrogels, several issues remain to be addressed: natural variations among batches of balsa wood; the appropriate safe dosage range for Zn^2+^; and the lack of confirmation of wound dressing efficacy through large animal studies. Future research will focus on: (1) establishing standardized, reproducible preparation protocols for wood materials (including density ranges, reaction temperatures, delignification levels, etc.); (2) determining Zn^2+^ release kinetics under simulated exudate flow conditions to establish a safe dosage range; and (3) conducting standardized wound healing test reports using relevant large animal partial-thickness wound models prior to any clinical translation evaluation.

## Figures and Tables

**Table 1:** Proportion of each component in the BWSM/PVA/CS/ZnG hydrogel dressing.

Ingredient	Proportion				
	AH-0	AH-1	AH-2	AH-3	AH-4
PVA	5	5	5	5	0
CS	2	2	2	0	0
ZnG	1	0	1	1	0
BWSM	No	Yes	Yes	Yes	Yes

**Table 2:** Heat loss weight data of balsa wood before and after treatment.

Sample	T_20%_ (°C)	T_50%_ (°C)	T_max_ (°C)	ω_m_ (%)
BW	282.9	319.3	318.7	22.9
BWS	279.6	335.5	346.9	14.3
BWSM	293.7	343.7	356.7	13.6

**Table 3:** Mass loss rate of balsa wood.

Sample	BW (g)	BWN (g)	Mass Loss Rate (%)
1	0.41	0.28	31.7
2	0.42	0.28	33.3
3	0.42	0.27	35.7

**Table 4:** BET analysis of dual-treated balsa wood.

Sample	BET Specific Surface Area (m^2^/g)	BET Adsorption Pore Size (nm)
BWS	8.0 ± 0.2	5.8 ± 0.07
BWSM	5.8 ± 0.17	6.3 ± 0.26

**Table 5:** Thermal weight loss data sheet for the AH hydrogel dressing.

Sample	T_20%_ (°C)	T_50%_ (°C)	T_max_ (°C)	ω_m_ (%)
AH-0	248.7	292.1	283.0	23.1
AH-1	248.2	259.4	278.3	19.9
AH-2	250.6	308.5	279.4	21.3
AH-3	274.8	307.8	289.2	15.6
AH-4	293.7	343.7	356.7	13.6

## Data Availability

The original contributions presented in this study are included in the article. Further inquiries can be directed to the corresponding authors.

## Abbreviations

The following abbreviations are used in this manuscript:

PVA	Polyvinyl Alcohol
CS	Chitosan
ZnG	Zinc Gluconate
BW	Balsa wood
BWN	Balsa wood treated with NaOH and Na_2_SO_3_
BWS	BWN treated with H_2_O_2_ aqueous solution
BWSM	BWS treated with Microwave
AH-0	PVA–CS–ZnG ratio of 5:2:1
AH-1	For cases with BWSM, the ratio of PVA–CS–ZnG is 5:2:0.
AH-2	For cases with BWSM, the ratio of PVA–CS–ZnG is 5:2:1.
AH-3	For cases with BWSM, the ratio of PVA–CS–ZnG is 5:0:1.
AH-4	Only BWSM
CS-3	PVA–CS–ZnG ratio of 5:3:1
Zn-3	PVA–CS–ZnG ratio of 5:2:3
T_20%_	Temperature at which 20% mass loss occurs during thermal stability test
T_50%_	Temperature at which 50% mass loss occurs during thermal stability test
T_max_	Temperature at the maximum weight loss rate during the thermal stability test
ω_m_	Mass residue rate at the end of the thermal stability test
${OD}_{ex}$	the optical density of the experimental group
${OD}_{b}$	the optical density of the blank background group
${OD}_{n}$	the optical density of blank control group
FTIR	Fourier transform infrared spectroscopy
XPS	X-ray Photoelectron Spectroscopy
DSC	Differential scanning calorimeter
TGA	Thermogravimetric analyzer
BET	Brunauer–Emmett–Teller
